# Nematic liquid crystal alignment on subwavelength metal gratings

**DOI:** 10.3762/bjnano.9.6

**Published:** 2018-01-04

**Authors:** Irina V Kasyanova, Artur R Geivandov, Vladimir V Artemov, Maxim V Gorkunov, Serguei P Palto

**Affiliations:** 1 Liquid crystals laboratory, Shubnikov Institute of Crystallography of Federal Scientific Research Centre “Crystallography and Photonics” of Russian Academy of Sciences, 119333 Leninsky Ave. 59, Moscow, Russia

**Keywords:** alignment, Fourier analysis, nematic liquid crystal, subwavelength metal grating

## Abstract

We have studied the alignment of a nematic liquid crystal (LC) material on aluminum subwavelength nanogratings as a function of the period, *p*, and the slit width to period ratio, *w/p*. A method, based on Fourier analysis of the transmittance spectra of the LC grating system, has been applied. We show that the gratings provide stable planar alignment only for shorter periods and narrower slits (*p* < 400 nm, *w*/*p* < 2/3). As these parameters increase, the homogeneous surface alignment changes to domains with different tilt angles or to spatially modulated alignment. We have also obtained a 90° twisted LC director distribution, implying sufficiently strong azimuthal LC anchoring at the grating surface.

## Introduction

In the age of nanotechnology, various nanostructured materials are under intensive investigation, being considered as perspective research objects for various applications [1–2]. Metallic materials with structures on a scale smaller than the visible wavelength are of special interest for different optical applications due to the existence of many types of resonances which make such materials potentially useful in optical filters, biochemical sensors, light polarizers and other devices [3–8]. Because liquid crystals (LCs) allow for the control of such resonances using external electric and magnetic fields, the idea of combining a nanomaterial with a liquid crystal into a hybrid system is especially interesting as it can result in even more novel and interesting properties. In our recent work, we showed that liquid crystals strongly affect both the plasmon resonance and light polarization properties of subwavelength metal gratings [9]. Meanwhile, these gratings can also be used as nanoelectrodes, which allow the LC to be driven by an electric field, enabling a very fast electro-optical effect due to the influence of the adjacent liquid crystal layer on the plasmonic resonance [10]. In all of these effects, the alignment of LC molecules on a metallic grating is of principal significance. Thus, the detailed study of the alignment of the liquid crystal at the grating surface is required, and this work shows our first results in this direction.

This paper consists of two principal parts. First, we briefly describe the sample preparation, which involves the ion-beam milling technique and assembly of the experimental liquid crystal cell. The second section is dedicated to data analysis, where besides polarized microscope observations, we apply the Fourier transform technique to the transmittance spectra in order to extract the effective values of the LC extraordinary refractive index. This information allows us to determine the average alignment of the LC molecules on the nanograting surface.

## Experimental

**Figure 1:**
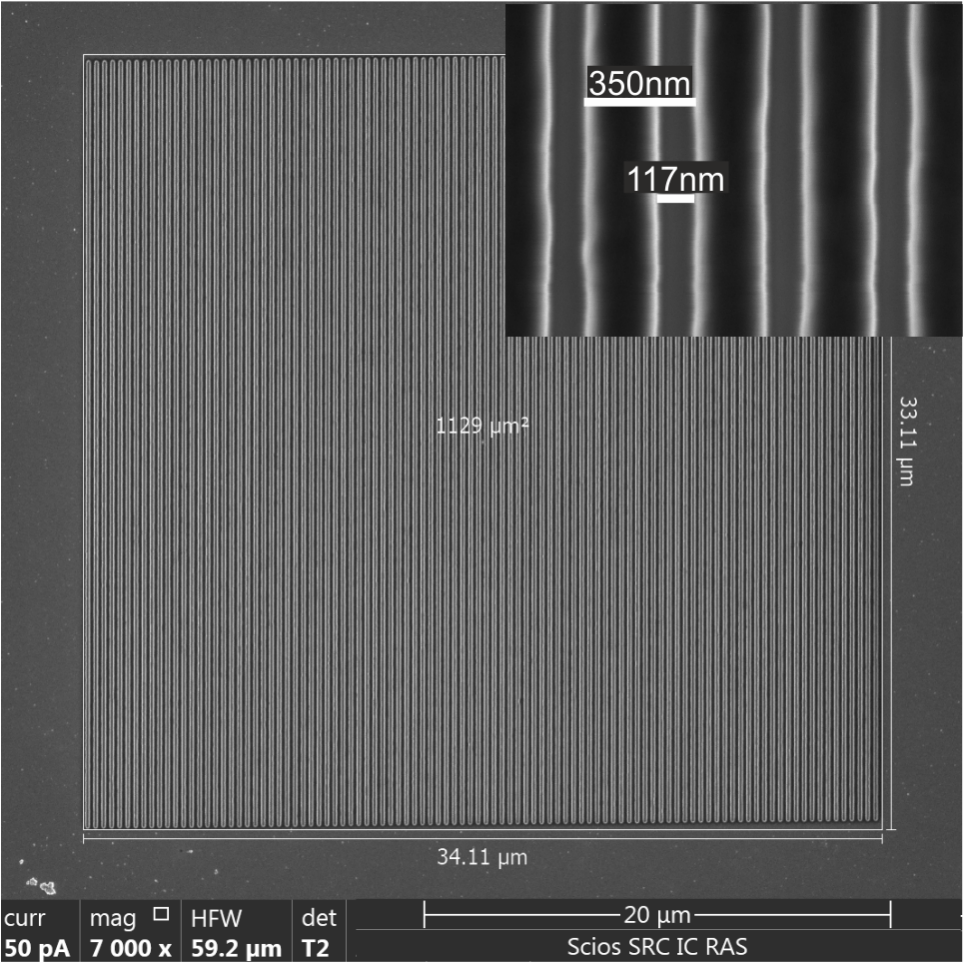
Electron microscope image of a grating with period *p* = 350 nm, duty factor *w*/*p* = 1/3.

We have fabricated aluminum films of ≈100 nm thickness on a glass substrate using the vacuum sputtering technique. The subwavelength gratings (Figure 1) are produced by ion-beam milling of the films using an FEI Scios dual beam electron-ion microscope (accelerating voltage 30 kV, ion beam current 0.1–0.3 nA).

**Figure 2:**
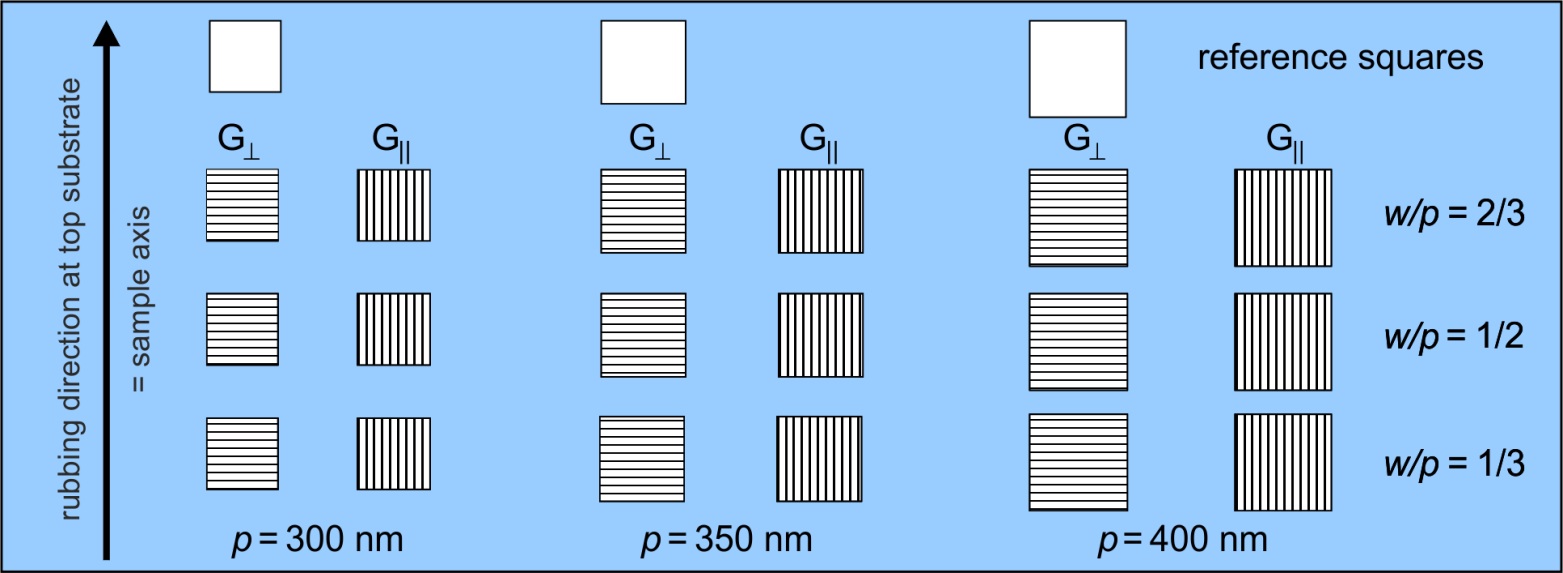
Schematic layout of the gratings on the sample; the arrow on the left indicates the rubbing direction of the top substrate to which the orientation of the grating slit is related.

We have produced a series of gratings on the same substrate in order to be able to observe the influence on LC alignment of such parameters as a function of grating period (*p*), and slit width (*w*) (Figure 2). The gratings were made with three periods: 300, 350, and 400 nm, with three different values of the duty factor (*w*/*p*) for each period. Furthermore, each of the nine geometries was made with two orthogonal directions of the grating slits. Since every grating contains 100 slits, and its size depends on the period, the reference squares of a size corresponding to each period were also milled. These squares, which are free of Al, were used as the reference areas in the optical spectra measurements.

The glass plate with the aluminum gratings was used as a substrate for a LC cell: it was stacked with another substrate, namely a glass plate covered with rubbed polyimide layer (to provide the planar LC alignment) in a way that for half of the gratings, the rubbing direction is perpendicular to the slits of the gratings, and parallel to the other half (the rubbing direction is depicted with an arrow on the left in Figure 2). The gratings will be referred to hereafter as “
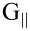
” if the slits are parallel to the top substrate rubbing direction (also called the "sample axis"), and “
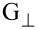
” if they are perpendicular to it. The gap between the glass plates was insured with teflon spacers and was measured to be 5.7 ± 0.5 μm. The cell is filled with Merck E7 LC material (*n*_o_ = 1.527, *n*_e_ = 1.751 at a wavelength of 546 nm and temperature *T* = 25.0 °C [11]).

## Results and Discussion

**Figure 3:**
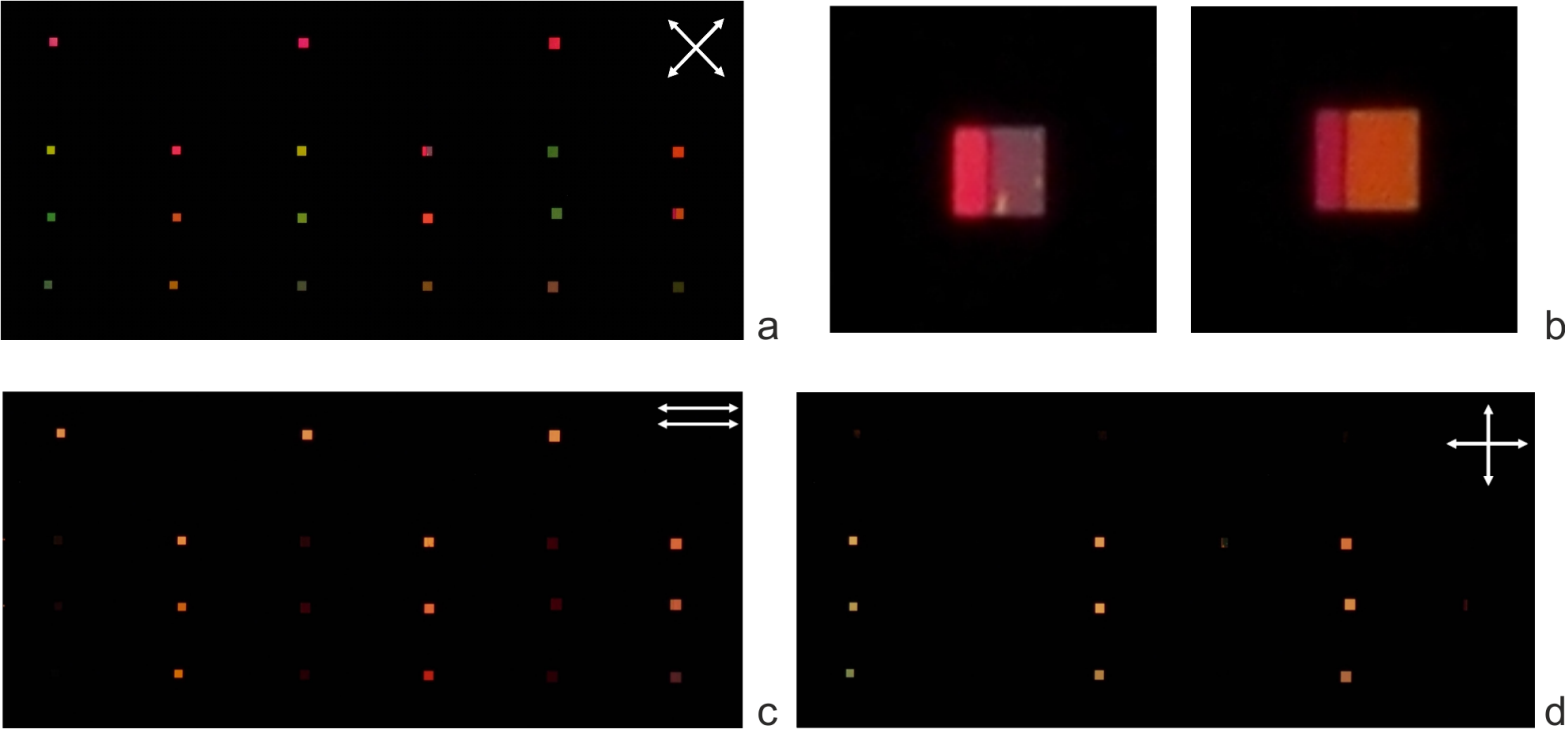
Photos of the liquid crystal cell under polarized light: (a) 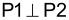, the sample axis (rubbing direction) is at 45° to P1 and P2; (b) the enlarged part of photo a, showing two 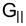 gratings: *p* = 350 nm, *w*/*p* = 2/3 (left) and *p* = 400 nm, *w*/*p* = 1/2; (c) P1 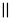 P2, the sample axis is at 90° to the input polarization P1; (d) 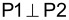, the sample axis is at 90° to the input polarization P1.

We performed visual observations of the cell under polarized light. In crossed polarizers with the sample axis at 45° to the polarizer axis (P1 and P2, Figure 3a) all the gratings and reference squares are bright. They provide different phase retardation and transmittance spectra as observed by their different colors in Figure 3.

Moreover, in some cases, different colors are formed within one grating (Figure 3b), which points out the difference in optical retardation and the LC alignment. When the polarizer axes are parallel (Figure 3c), the reference squares and 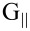 gratings are bright and the 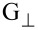 gratings are dark. In the crossed polarizers with the sample axis perpendicular to the input polarization (Figure 3d), on the contrary, 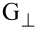 gratings are bright and 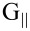 gratings and the reference squares are dark. Such a behavior proves that the LC director above the 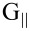 gratings is aligned in the plane formed by the sample axis and the normal to the gratings, whereas above the 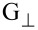 gratings, a twisted distribution of the LC director takes place in the layer. The appearance of the twist demonstrates that the subwavelength aluminum gratings are capable of aligning liquid crystals along their slits with rather small pretilt angles and that the anchoring energy is high enough to balance the elastic torque at the surface, which is provided by the twisted deformation across the LC layer. The azimuthal anchoring strength can be estimated from the fact that the angular deviation from the 90^o^ twist is not higher than our experimental accuracy (δφ ≈ 3°). Our analysis shows that in the case of such small deviations the lowest value for the anchoring energy magnitude can be estimated as *W*_0_ ≥ π*K_2_*/(2*d* δφ) *=* 2.6 × 10^−2^ mJ/m^2^, where we use *K_2_* 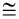 5 pN , *d* = 6 μm, and δφ = 0.05 radians. The estimated value is of the same order of the values as for the azimuthal anchoring strength for the E7 on the polyimide surface [12].

**Figure 4:**
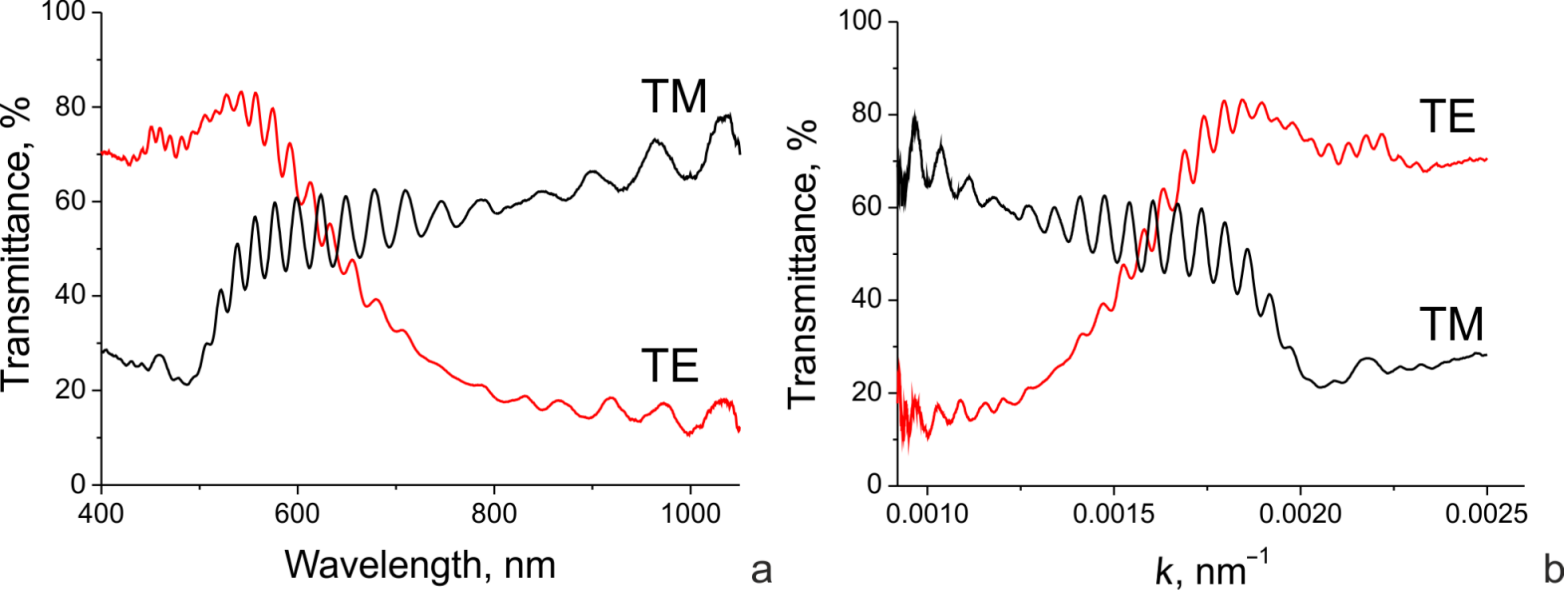
The transmittance for the grating with *p* = 300 nm and *w*/*p* = 1/2 versus wavelength (a) and wavenumber (b).

In order to gain a better understanding of the pretilt angle of the LC director at the grating surface, we have analyzed the transmittance spectra (Figure 4). These spectra are measured with respect to the reference squares using an Avantes AvaSpec-2048-USB2-UA optical fiber spectrometer mounted on an Olympus CX31PF-5 (equipped with U-CTR 30-2 trinocular tube) polarized microscope. Besides the resonance performance, resulting in high transmittance and dips in the range of 400–550 nm (the spectral range of the resonance transmittance depends on the grating geometry, which was discussed in detail in our work [9]), the spectra show pronounced fine oscillations caused by the Fabry–Perot effect. It is the Fabry–Perot effect that is used here to extract some information on the LC alignment near the grating surface. It is useful for further consideration to present the spectra as transmittance vs wavenumber (*k* = 1/λ) (Figure 4b). In this case, the almost constant period of the fine oscillations is more obvious.

It follows from the simplest Fabry–Perot model considering only single reflections from each boundary that the contribution to the transmittance is:

[1]
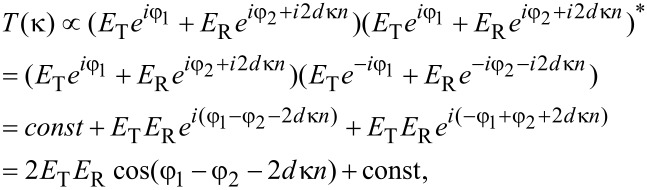


where "*" designates the complex conjugation, 
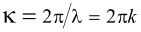
 is the angular wavenumber and *n* is the refraction coefficient of the layer between the reflecting boundaries. *E*_T_ and φ_1_ are amplitude and phase of the first (incident) wave, respectively, at the output boundary, and *E*_R_ and φ_2_ are the corresponding characteristics of the second wave, reflected first from the output and then from the input boundary before it leaves the layer. In the general case φ_1_ ≠ φ_2_, following from Equation 1, the condition for transmittance maxima is

[2]
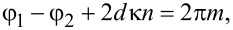


where *m* is an integer. Thus, the maxima wavenumber is defined as

[3]
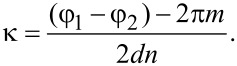


For two neighbor maxima, the change of *m* is unity (Δ*m* = 1), and if we neglect the spectral dispersion inside a spectral interval Δκ then the spectral interval between the two neighbor maxima is

**Figure 5:**
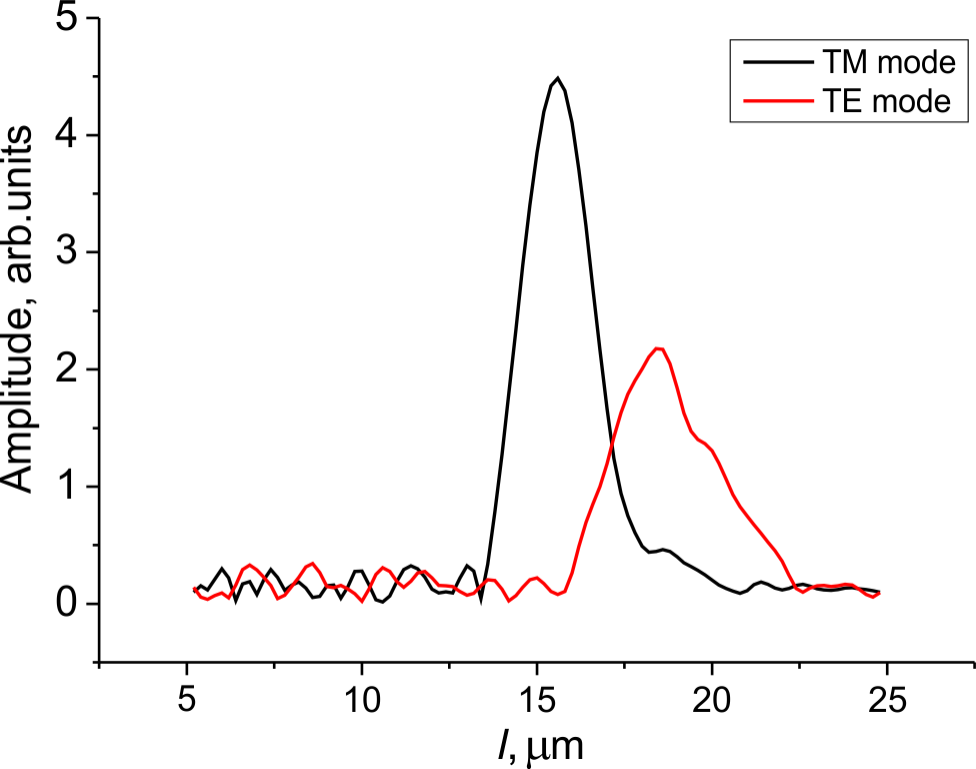
The Fourier transform spectra of the transmittance spectra given in Figure 4b (grating *p* = 300 nm, *w*/*p* = 1/2).

[4]
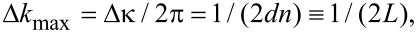


where 2*dn =* 2*L* is twice the optical thickness of the layer. Thus, the optical thickness can be extracted from the transmittance spectra by measuring a period of the oscillations. Instead of directly finding an average period of the oscillations in the spectra, one can use the Fourier transform (FT) of the spectral data given in Figure 4b, which minimizes the error. The choice of the spectral window is important for this method. The rectangular window implied in the very idea of the spectral acquisition technique is not the best choice in this case due to the rather narrow spectral range of the measured spectra and the high impact of the spectral window edges (the effect is similar to optical diffraction from a narrow rectangular slit). In order to avoid undesired components in the FT spectra we used a Gaussian window with the following parameters: *k*_max_ = 0.00183 nm^−1^ (λ = 546 nm), Δ*k* = 0.0004 nm^−1^ (in the wavelength range, the full-width at half-maximum is about 120 nm). The resulting Fourier transform spectra have pronounced maxima corresponding to *l =* 2*L* (Figure 5). The width of the band in the FT spectrum is defined by the spectral dispersion of the refractive index and the whole spectral interval of the original transmittance spectrum due to the spectral window width.

The different positions of the two maxima related to the transverse electric (TE) and transverse magnetic (TM) modes are due to the optical anisotropy of the LC: the TM mode is polarized along the grating wavevector (and across the slits), thus, it deals with the ordinary refractive index, *n*_o_, whereas the orthogonal TE mode interacts with the extraordinary refractive index, *n*_e_.

Based on the FT spectrum of the TM mode, and taking into account the value of the ordinary refractive index known with a high accuracy (*n*_o_ = 1.527 at 546 nm [10]) together with its low spectral dispersion (d*n*_o_/dλ ≈0.0001 nm^−1^), an accurate value of the local LC layer thickness can be evaluated as *d*_local_ = *L*_TM_/2*n*_o_ = 5.1 ± 0.05 μm. The inaccuracy is defined by spectral dispersion of the ordinary refractive index. Then the effective extraordinary index can be found from the TE spectrum maximum: *n*_e_ = *L*_TE_/2*d*_local_ = 1.73 ± 0.01. Given the inaccuracy, the found value of the effective extraordinary index is very close to the principal refractive index corresponding to the dielectric permittivity tensor component along the LC director (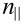 = 1.751 at 546 nm). Thus, we can conclude that for the given short-period grating we achieve a quite good planar alignment when the pretilt angle (in the cases where it exists) is very low (our inaccuracy corresponds to a few degrees).

**Figure 6:**
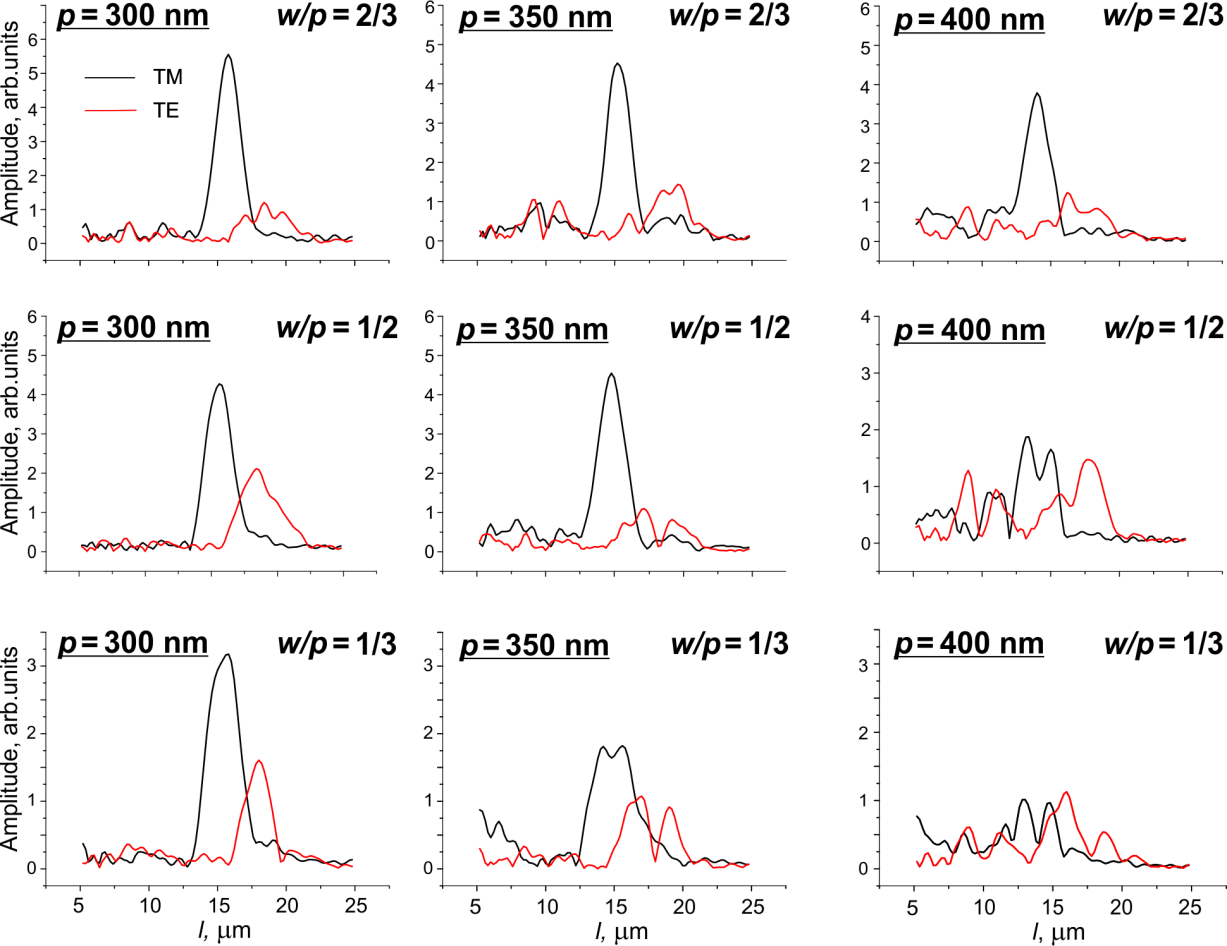
Fourier transform spectra vs grating geometry.

The Fourier transform spectra are quite distinct for smaller periods and the narrowest slits (*p* = 300 nm, *w*/*p* = 1/2 and *w*/*p* = 1/3), indicating single maxima exist for both TE and TM polarizations. Meanwhile, the other geometries are less clear, and the maximum tends to split as the period and duty factor increase (Figure 6).

There are two reasons for the band splitting in the FT spectra. The first one is related to the spectral dispersion of the principal refractive indices. Let us consider, for example, the grating with *p* = 350 nm and *w*/*p* = 1/3. For this grating both TM and TE-mode FT spectra show pronounced splitting. However, it is known [9] that for the gratings with this geometry there is a plasmon resonance for the TM mode and the transmittance resonance for the TE mode in the spectral range of 500–600 nm. Therefore, the Fabry–Perot effect is suppressed in this spectral range and, actually, we cut the whole measured spectral range for the two fractions: i) 400–500 nm and ii) 600–1000 nm. Due to spectral dispersion of the principal refractive indices, for the average values in the given spectral intervals we have *n*_o,400–500_ 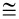 1.53 and *n*_o,600–1000_ 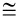 1.52. The corresponding splitting, δ*l*_o_, in the FT spectrum for the TM mode is 2δ*n*_o_*d*_loc_ 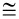 1000 nm, which is in a good agreement with that in the FT spectrum. The spectral dispersion for the extraordinary refractive index (*n*_e_) is higher (*n*_e_ 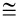 1.78 at λ = 480 nm and *n*_e_ 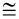 1.72 at λ = 700 nm), which explains the higher splitting (δ*l*_e_ 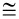 2500 nm) of the band in the FT spectrum of the TE mode. The idea of the spectral dispersion origin of the splitting is also supported by FT analysis using narrower spectral windows. A spectral shift of the window allows for increased impact of one of the two spectral ranges.

However, the spectral dispersion cannot explain all the results, especially for the gratings with *p* = 400 nm. Another possible reason can be the undulation of the LC director at the grating surface or the appearance of domains with different pretilt angles. Thus, we have to conclude that there is a hybrid or inhomogeneous alignment, as in the case shown in Figure 3b, where two domains with different pretilt angles coexist.

We present the results of Fourier analysis only for the 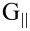 gratings; in the case of 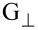 gratings, where the twisted alignment takes place, the input polarization is rotated by the twisted LC layer, which does not allow independent measurements of the TE and TM-mode spectra.

## Conclusion

We have demonstrated that the subwavelength aluminum nanogratings can be used to align nematic liquid crystal material with its easy axis along the slits. In order to study the alignment, the Fourier transform method was proposed. It was found that the homogeneous alignment near the gratings strongly depends on their geometry. For the shortest grating period and narrow slits, the alignment is found to be planar with a close to zero pretilt angle. The alignment on gratings with higher periods becomes unstable and shows either domains or undulations, resulting in the splitting and broadening of the bands in the Fourier transform spectra.

## References

[1] Aricò A S, Bruce P, Scrosati B, Tarascon J-M, van Schalkwijk W (2005). Nat Mater.

[2] Konstantatos G, Sargent E H (2010). Nat Nanotechnol.

[3] Genet C, Ebbesen T W (2007). Nature.

[4] Xu T, Wu Y-K, Luo X, Guo L J (2010). Nat Commun.

[5] Decker M, Zhao R, Soukoulis C M, Linden S, Wegener M (2010). Opt Lett.

[6] Gorkunov M V, Ezhov A A, Artemov V V, Rogov O Y, Yudin S G (2014). Appl Phys Lett.

[7] Tang Y, Cohen A E (2010). Phys Rev Lett.

[8] Wang Y, Xu J, Wang Y, Chen H (2013). Chem Soc Rev.

[9] Palto S P, Barnik M I, Artemov V V, Shtykov N M, Geivandov A R, Yudin S G, Gorkunov M V (2015). J Appl Phys.

[10] Palto S P, Barnik M I, Kasyanova I V, Geivandov A R, Shtykov N M, Artemov V V, Gorkunov M V (2016). JETP Lett.

[11] Li J, Wen C-H, Gauza S, Lu R, Wu S-T (2005). J Disp Technol.

[12] Acharya B R, Kim J-H, Kumar S (1999). Phys Rev E.

